# Supplementary material for: Selective attenuation of Ether-a-go-go related K+ currents by endogenous acetylcholine reduces spike-frequency adaptation and network correlation
Source: eLife. 2019 Apr 29;8:e44954. doi: 10.7554/eLife.44954 (PMC6488300; doi:10.7554/eLife.44954)
Supplement: Transparent reporting form [file elife-44954-transrepform.pdf]

eLIFE
1st Floor
24 Hills Road
Cambridge CB2 1JP, UK
P 01223 855340
W elifesciences.org
T @elife

eLife's transparent reporting form

We encourage authors to provide detailed information *within their submission* to facilitate the interpretation and replication of experiments. Authors can upload supporting documentation to indicate the use of appropriate reporting guidelines for health-related research (see EQUATOR Network), life science research (see the BioSharing Information Resource), or the ARRIVE guidelines for reporting work involving animal research. Where applicable, authors should refer to any relevant reporting standards documents in this form.

If you have any questions, please consult our Journal Policies and/or contact us: editorial@elifesciences.org.

Sample-size estimation

- You should state whether an appropriate sample size was computed when the study was being designed
- You should state the statistical method of sample size computation and any required assumptions
- If no explicit power analysis was used, you should describe how you decided what sample (replicate) size (number) to use

Please outline where this information can be found within the submission (e.g., sections or figure legends), or explain why this information doesn't apply to your submission:

Most of the experiments in this report typically involve detecting differences between means of 2 different groups at the 95% confidence level. In general, our typical data yielded responses within groups that were normally distributed. We always tested explicitly for a normal distribution before applying tests dependent on a normal distribution (e.g., t-tests). We used alternate statistical tests if we found non-normal distributions. In a typical power analysis, we determined that a total sample size of 4-5 (experimental and control cells) to be able to reject the null hypothesis that population means in the experimental and control groups are equal with a power of 0.8. The type 1 error probability associated with this test of the null hypothesis is 0.05.

Replicates

- You should report how often each experiment was performed
- You should include a definition of biological versus technical replication
- The data obtained should be provided and sufficient information should be provided to indicate the number of independent biological and/or technical replicates
- If you encountered any outliers, you should describe how these were handled
- Criteria for exclusion/inclusion of data should be clearly stated
- High-throughput sequence data should be uploaded before submission, with a private link for reviewers provided (these are available from both GEO and ArrayExpress)

Please outline where this information can be found within the submission (e.g., sections or figure legends), or explain why this information doesn't apply to your submission:

Most statistical comparisons were based on experimental data collected over 7-10 days using the same apparatus and under the same conditions. All Ns for statistical tests are indicated in the manuscript (mostly in either the figures or figure legends). All replicates in this study were biological (except for the computational simulation results). The Methods section in the manuscript contains the parameters used to define healthy neurons for inclusion in the study. No outliers were excluded when conducting tests.

Statistical reporting

- Statistical analysis methods should be described and justified
- Raw data should be presented in figures whenever informative to do so (typically when N per group is less than 10)
- For each experiment, you should identify the statistical tests used, exact values of N, definitions of center, methods of multiple test correction, and dispersion and precision measures (e.g., mean, median, SD, SEM, confidence intervals; and, for the major substantive results, a measure of effect size (e.g., Pearson's r, Cohen's d)
- Report exact p-values wherever possible alongside the summary statistics and 95% confidence intervals. These should be reported for all key questions and not only when the p-value is less than 0.05.

Please outline where this information can be found within the submission (e.g., sections or figure legends), or explain why this information doesn't apply to your submission:

For each statistical test used we explicitly state which type of test was used (e.g., paired t-test), the Ns, the means, the SEMs, the P value and T value. In general, the tests we applied were the most appropriate for small sample sizes with normally distributed data. All t-tests were adjusted for multiple comparisons using the Bonferroni method, as indicated in the Methods section. We believe these parameters completely describe the statistical results we found. For most tests we include these parameters in the figure legends.

For large datasets, or papers with a very large number of statistical tests, you may upload a single table file with tests, Ns, etc., with reference to sections in the manuscript.)

Group allocation

- Indicate how samples were allocated into experimental groups (in the case of clinical studies, please specify allocation to treatment method); if randomization was used, please also state if restricted randomization was applied
- Indicate if masking was used during group allocation, data collection and/or data analysis

Please outline where this information can be found within the submission (e.g., sections or figure legends), or explain why this information doesn't apply to your submission:

Not applicable in this type of study.

Additional data files ("source data")

- We encourage you to upload relevant additional data files, such as numerical data that are represented as a graph in a figure, or as a summary table
- Where provided, these should be in the most useful format, and they can be uploaded as "Source data" files linked to a main figure or table
- Include model definition files including the full list of parameters used

- Include code used for data analysis (e.g., R, MatLab)
- Avoid stating that data files are "available upon request"

Please indicate the figures or tables for which source data files have been provided:

Not applicable for this type of study where the raw data is intracellular recordings. We would be happy to provide the Python and Matlab analysis routines used to readers upon request.

eLife Sciences Publications, Ltd is a limited liability non-profit non-stock corporation incorporated in the State of Delaware, USA, with company number 5030732, and is registered in the UK with company number FC030576 and branch number BR015634 at the address 1st Floor, 24 Hills Road, Cambridge CB2 1JP | August 2014
